# VEGFR-2 inhibitors and apoptosis inducers: synthesis and molecular design of new benzo*[g]*quinazolin bearing benzenesulfonamide moiety

**DOI:** 10.1080/14756366.2017.1334650

**Published:** 2017-06-29

**Authors:** Mostafa M. Ghorab, Mansour S. Alsaid, Aiten M. Soliman, Fatma A. Ragab

**Affiliations:** a Department of Pharmacognosy, College of Pharmacy, King Saud University, Riyadh, Saudi Arabia;; b Department of Drug Radiation Research, National Center for Radiation Research and Technology, Egyptian Atomic Energy Authority, Cairo, Egypt;; c Department of Pharmaceutical Chemistry, Faculty of Pharmacy, Cairo University, Cairo, Egypt

**Keywords:** Benzo[g]quinazolin, sulfonamide, VEGFR-2 inhibitors, apoptosis, breast cancer

## Abstract

Two series of novel 4-(2-(2-(2-(substituted) hydrazinyl)-2-oxoethylthio)-4-oxobenzo[*g*]quinazolin-3(*4H*)-yl) benzenesulfonamide **5–17** and 4-(2-(2-(substituted-1*H*-pyrazol-1-yl)-2-oxoethylthio)-4-oxobenzo[*g*]quinazolin-3(4*H*)-yl) benzenesulfonamide **18–24** were synthesised from the starting material 4-(2-(2-hydrazinyl-2-oxoethylthio)-4-oxobenzo[*g*]quinazolin-3(*4H*)-yl) benzenesulfonamide **5,** to be evaluated for their inhibitory activity towards VEGFR-2. The target compounds **5–24,** were screened for their cytotoxic activity against MCF-7 breast cancer cell line and the percentage inhibition against VEGFR-2. Compounds **9, 20, 22** and **23**, showed excellent VEGFR-2 inhibitory activity with IC_50_ ranging from 0.64 to 1.04 µm. Being the most potent, compound **9** was evaluated for its apoptotic inducer effect by studying the effect on caspase-3, it was found to increase its level. Compound **9** boosted the level of Bax and reduced the level of BCl2, compared to the control. Cell cycle analysis was conducted, compound **9** showed cell cycle arrest at G2/M phase. Moreover, mild cytotoxic effect (IC_50_ = 29.41 µm, respectively) in normal breast cells MCF-12 A, was observed when treated with the same compound. Finally, a molecular docking study was performed to investigate the possible binding interaction inside the active site of the VEGFR-2 enzyme.

## Graphical Abstract

**Figure:**
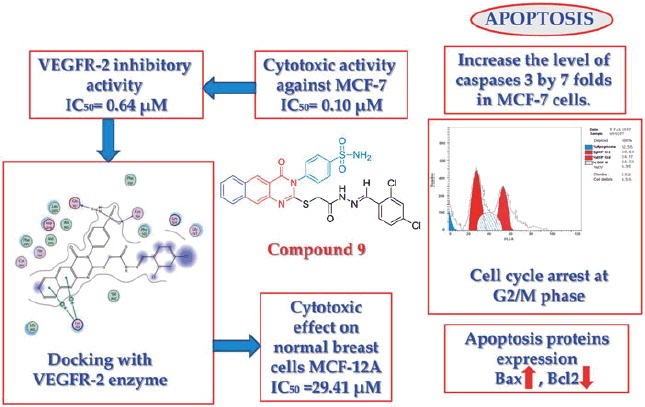

## Introduction

**Figure 1.**
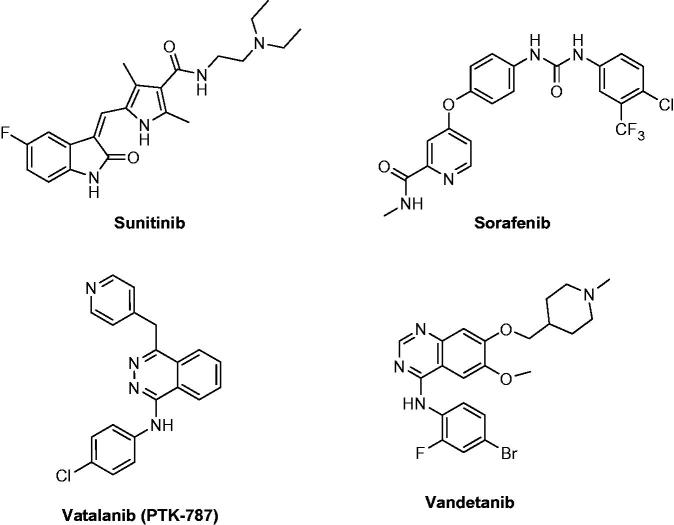
Structures of VEGFR inhibitors.

Tyrosine kinases are responsible for the phosphorylation of tyrosine residues in proteins. This phosphorylation leads to changing the protein’s function^1^. They are considered an important member involved in cell signalling pathways^2^. Mutations can cause some tyrosine kinases to become continuously active, leading to the development of cancer^3^. Vascular endothelial growth factor (VEGF) is an important signalling protein involved in both vasculogenesis and angiogenesis^4^. It was found to enhance the microvascular permeability thus, promoting endothelial cell mitogenesis and cell migration^5^. VEGF is up-regulated in many tumours due to an imbalance between proangiogenic and anti-angiogenic factors^6^. They consist of three subtypes, which are VEGFR-1 (Flt-1), VEGFR-2 (KDR) and VEGFR-3 (Flt-4)^7^. Selectivity of kinase inhibitors is difficult to predict based on chemical structure and sequence. VEGFR-2 can be divided into three subtypes, kinase I inhibitors, which interact with the ATP-binding site by one to three hydrogen bonds, mimic that formed by ATP^8^. An example of this type is sunitinib (Figure 1)^9^, which demonstrated competitive inhibition to ATP. On the contrary, type II indirectly competes with ATP by occupying the hydrophobic pocket adjacent to the ATP binding site. Some type II inhibitors are able to form hydrogen bonds to the ATP binding site. However, this is not necessary for activity^10^. An example of type II inhibitors is sorafenib (Figure 1) that acts by blocking the phosphorylation of VEGFR by using its hydrophobic pocket^11^. The third class is known as covalent inhibitors, they covalently bind to cysteine at specific sites of the kinase allowing the inhibitor to block the binding of ATP to the kinase^12^. An example of these inhibitors is Vatalanib (Figure 1)^13^. Vandetanib, the reference drug used in this study is a dual inhibitor towards VEGFR-2 and EGFR, approved in 2011, it was found to disrupt the angiogenesis process and starve tumours of nutrients. It is an example of an extended-spectrum agent^14^.

Once activated, VEGFR-2 undergoes autophosphorylation, triggering signalling pathways leading to endothelial cell proliferation and subsequent tumour angiogenesis^15^^,^^16^. Folkman^17^ proposed that tumour growth and metastasis are angiogenesis-dependent, and hence, blocking angiogenesis could be a strategy to hinder tumour growth.

Breast cancer (BC) is the most common malignancy and the leading cause of cancer death in women worldwide^18^. VEGFR-2 inhibitors were reported in treating BC due to their high safety profile^19–21^. But a combination therapy with other chemotherapy or radiotherapy was reported to maximise the therapeutic effect^22^.

**Figure 2.**
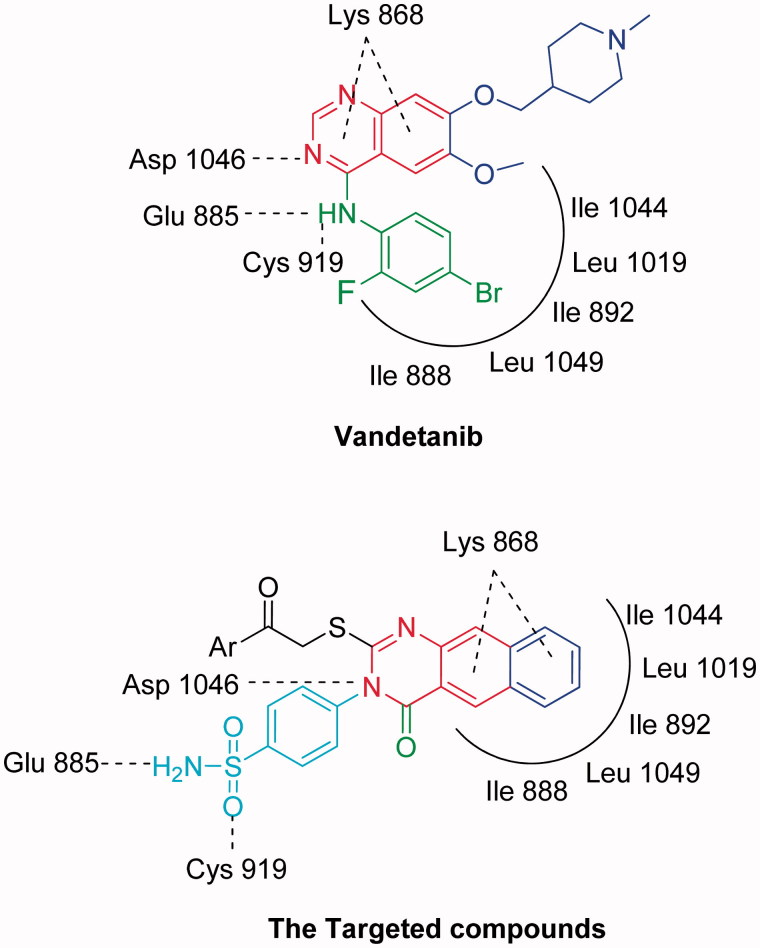
The design concepts of the targeted compounds.

In order to design our targeted compounds, the essential requirements for VEGFR-2 receptor bearing quinoline inhibitor (PDB ID: 3U6J) were studied^23–25^ (Figure 2): (i) Fused aromatic ring system represented by a quinoline ring interacting as hydrogen bond acceptor by its nitrogen atom with Asp 1046 and form hydrophobic interactions with Lys 868. This quinoline ring was replaced by benzo[*g*]quinazoline in our target structures and forms the same interactions. (ii) Substituted anilino group in position 4 of the quinoline interacting as an H-bond donor by its NH with Glu885 and through hydrophobic interaction by its substituted phenyl moiety with the hydrophobic back pocket lined with the hydrophobic side chains of Ile 888, Ile 892, Leu 1019 and Ile 1049. In our target compounds, the substituted anilino group was replaced with NH_2_ of the sulfonamide group and was found to form H-bond with Glu 885, while, the benzo[*g*]quinazolin fits inside the hydrophobic pocket. (iii) Hydrogen bond acceptor as nitrogen lone pair or oxygen atom attached to position 4 of quinoline via benzyl or phenyl moiety, which interacts with the backbone NH of Cys 919 in the hinge region of the enzyme. In our target compounds, the oxygen atom in the sulfonamide group binds with Cys 919.

In this respect, we designed novel compounds based on benzo[*g*]quinazoline core and sulfonamide moiety, these derivatives were subjected to *in vitro* cytotoxic evaluation against MCF-7, followed by VEGFR-2 inhibitory profile. Molecular docking was performed in the active site of VEGFR-2, to determine their binding mode and their ability to satisfy the pharmacophoric features required to induce the desired inhibition. Moreover, VEGFR-2 inhibition in cancer cells was found to trigger apoptosis which synergistically augments the antitumour effect^26^^,^^27^. So, the apoptotic effect of the most potent compound was discussed in comparison to vandetanib, through the inhibition of the caspase-3 enzyme, detection of BAX and BCl2 levels, and cell cycle analysis. Also, the cytotoxicity of the most potent compound against normal breast cell line was investigated.

## Materials and methods

Melting points were determined in an open capillary on a Gallen Kamp melting point apparatus (Sanyo Gallen Kamp, Leicestershire, UK). Precoated aluminium sheets Silica gel Merck 60 F254 were used for thin layer chromatography and were visualised by UV lamp (Merck, Darmstadt, Germany). The developing solvent system was chloroform/methanol 7:3. IR spectra (KBr disc) were recorded using an FT-IR spectrophotometer (PerkinElmer), OH, USA. ^1^H-NMR spectra were scanned on an NMR spectrophotometer (Bruker AXS Inc., Flawil, Switzerland), operating at 500 MHz for ^1^H- and 125.76 MHz for ^13^C. Chemical shifts are expressed in δ-values (ppm) relative to trimethylsilyl group as an internal standard, using DMSO-d_6_ as a solvent. Elemental analyses were done on a model 2400 CHNSO analyzer (PerkinElmer). All the values were within ±0.4% of the theoretical values. All reagents used were of AR grades.

## Chemistry

### 4-(2-Mercapto-4-oxobenzo*[g]*quinazolin-3*(4H)*-yl) benzenesulfonamide **(3)**

A mixture of 3-amino-2-naphthoic acid **1** (1.87 g, 0.01 mol) and 4-isothiocyanatobenzenesulfonamide **2**^28^ (2.14 g, 0.01 mol) in absolute ethanol (30 ml) containing 3 drops of triethylamine, was refluxed for 2 h. The solid obtained was filtered and crystallised from ethanol to give **3**.

Yield, 92%; m.p. 211–213 °C. IR (KBr) cm^−1^: 3390, 3278 (m, NH_2_ stretch), 3068 (s, CH arom. stretch), 1703 (s, CO stretch), 1633 (m, CN stretch), 1357, 1159 (m, SO_2_ stretch). ^1^H NMR (DMSO-d_6_, 500 MHz)δ: 7.5–8.1 (m, 10H, Ar-H), 8.7 (s, 2H, SO_2_NH_2_), 13.2 (s, 1H, SH). ^13^C NMR (DMSO-d_6_, 125.7 MHz)δ: 111.8, 116.5 (2), 126.3, 126.9, (2), 127.8, 129.8 (2), 130.0, 130.1, 130.2, 130.5, 135.7, 136.7, 144.1, 160.3, 176.0. MS *m/z* (%): 383 (M^+^) (9.22), 226 (100). Anal. Calcd. for C_18_H_13_N_3_O_3_S_2_ (383.44): C, 56.38; H, 3.42; N, 10.96. Found: C, 56.05; H, 3.25; N, 10.79.

### Ethyl-2-(4-oxo-3-(4-sulfamoylphenyl)-3,4-dihydrobenzo*[g]*quinazolin-2-ylthio)acetate **(4)**

A mixture of **3** (3.83 g, 0.01 mol) and ethyl chloroacetate (1.24 g, 0.01 mol) in dry acetone (50 ml) and anhydrous K_2_CO_3_ (2 g) was refluxed for 24 h. The solid obtained was filtered and crystallised from ethanol to give **4**. Yield, 88%; m.p. 147–149 °C. IR (KBr) cm^−1^: 3429, 3312 (m, NH_2_ stretch), 3100 (s, CH arom. stretch), 2983, 2841 (s, CH aliph. stretch), 1749, 1691 (s, CO stretch), 1631 (m, CN stretch), 1344, 1159 (m, SO_2_ stretch). ^1^H NMR (DMSO-d_6_, 500 MHz)δ: 1.2 (t, 3H, CH_3_), 4.0 (s, 2H, CH_2_), 4.3 (q, 2H, CH_2_ ester), 7.5–8.8 (m, 10H, Ar-H), 9.7 (s, 2H, SO_2_NH_2_). ^13^C NMR (DMSO-d_6_, 125.7 MHz)δ: 14.4, 34.9, 61.3, 119.2, 119.7 (2), 123.4, 126.7 (2), 128.2, 128.5 (2), 129.0, 129.4, 131.0, 131.4, 136.8, 140.2, 142.8, 161.2, 167.9, 168.8. MS *m/z* (%): 469 (M^+^) (2.47), 312 (100). Anal. Calcd. for C_22_H_19_N_3_O_5_S_2_ (469.53): C, 56.28; H, 4.08; N, 8.95. Found: C, 56.54; H, 4.33; N, 9.28.

### 4-(2-(2-Hydrazinyl-2-oxoethylthio)-4-oxobenzo*[g]*quinazolin-3*(4H)*-yl)benzenesulfonamide **(5)**

A mixture of **4** (4.69 g, 0.01 mol) and hydrazine hydrate (1.0 g, 0.02 mol) in absolute ethanol (50 ml) was stirred at room temperature for 19 h. The reaction mixture was filtered and the obtained solid was crystallised from ethanol to give **5**. Yield, 94%; m.p. 219–221 °C. IR (KBr) cm^−1^: 3475, 3319, 3255 (m, NH_2_, NH stretch), 3057 (s, CH arom. stretch), 2983, 2939 (s, CH aliph. stretch), 1749, 1680 (s, CO stretch), 1627 (m, CN stretch), 1394, 1159 (m, SO_2_ stretch). ^1^H NMR (DMSO-d_6_, 500 MHz)δ: 4.0 (s, 2H, CH_2_), 4.5 (s, 2H, NH_2_), 7.5–8.2 (m, 10H, Ar-H), 8.8 (s, 2H, SO_2_NH_2_), 9.7 (s, 1H, NH). ^13^C NMR (DMSO-d_6_, 125.7 MHz)δ: 34.9, 119.3, 123.4 (2), 126.7, 128.2 (2), 128.8, 129.4 (2), 129.8, 131.0, 131.2, 131.4, 136.9, 140.1, 142.6, 161.2, 166.5, 168.8. MS *m/z* (%): 455 (M^+^) (43.21), 354 (100). Anal. Calcd. for C_20_H_17_N_5_O_4_S_2_ (455.51): C, 52.54; H, 3.76; N, 15.37. Found: C, 52.39; H, 3.99; N, 15.04.

### General procedure for the synthesis of compounds 6–12

A mixture of **5** (4.55 g, 0.01 mol) and aromatic aldehyde (0.01 mol) in n-butanol (15 ml) was refluxed for 6 h. The obtained solid was filtered and crystallised from dioxane to give **6–12**.

#### 4-(2-(2-(2-(2, 5-Dimethylbenzylidene)hydrazinyl)-2-oxoethylthio)-4-oxobenzo*[g]*quinazolin-3*(4H)*-yl)benzenesulfonamide **(6)**

Yield, 81%; m.p. 177–179 °C. IR (KBr) cm^−1^: 3454, 3391, 3219 (m, NH_2_, NH stretch), 3053 (s, CH arom. stretch), 2920, 2836 (s, CH aliph. stretch), 1691, 1681 (s, CO stretch), 1618 (m, CN stretch), 1350, 1157 (m, SO_2_ stretch). ^1^H NMR (DMSO-d_6_, 500 MHz)δ: 2.3 (s, 6H, 2CH_3_), 4.7 (s, 2H, CH_2_), 7.0–8.6 (m, 13H, Ar-H), 8.8 (s, 1H, CH), 10.1 (s, 2H, SO_2_NH_2_), 11.7 (s, 1H, NH). ^13^C NMR (DMSO-d_6_, 125.7 MHz)δ: 18.2, 21.0, 34.5, 118.6, 122.1 (2), 124.0, 126.0, 126.8 (2), 127.2 (2), 127.6, 127.9, 128.3, 128.8, 129.0, 129.4, 131.2, 132.3, 132.8, 134.7, 134.9, 135.8, 140.6, 143.1, 159.6, 164.8, 174.0. MS *m/z* (%): 571 (M^+^) (8.84), 466 (100). Anal. Calcd. for C_29_H_25_N_5_O_4_S_2_ (571.67): C, 60.93; H, 4.41; N, 12.25. Found: C, 60.58; H, 4.19; N, 12.01.

#### 4-(2-(2-(2-(3-Fluoro-4-methylbenzylidene)hydrazinyl)-2-oxoethylthio)-4-oxobenzo*[g]*quinazolin-3*(4H)*-yl)benzenesulfonamide **(7)**

Yield, 82%; m.p. 250–252 °C. IR (KBr) cm^−1^: 3460, 3391, 3234 (m, NH_2,_ NH stretch), 3061 (s, CH arom. stretch), 2970, 2927 (s, CH aliph. stretch), 1688, 1678 (s, CO stretch), 1625 (m, CN stretch), 1350, 1155 (m, SO_2_ stretch). ^1^H NMR (DMSO-d_6_, 500 MHz)δ: 2.2 (s, 3H, CH_3_), 4.2 (s, 2H, CH_2_), 7.0–8.2 (m, 13H, Ar-H), 8.8 (s, 1H, CH), 9.9 (s, 2H, SO_2_NH_2_), 11.5 (s, 1H, NH). ^13^C NMR (DMSO-d_6_, 125.7 MHz)δ: 14.7, 32.6, 115.0, 120.6 (2), 124.9, 126.2 (2), 126.7 (2), 126.9 (2), 127.0, 127.7, 129.0, 129.3, 132.2 (2), 132.9 (2), 136.7, 140.8, 142.6, 145.5, 160.3, 160.7, 163.9, 170.1. MS *m/z* (%): 575 (M^+^) (12.64), 149 (100). Anal. Calcd. for C_28_H_22_FN_5_O_4_S_2_ (575.63): C, 58.42; H, 3.85; N, 12.17. Found: C, 58.12; H, 3.51; N, 12.02.

#### 4-(2-(2-(2-(4-Hydroxy-3-methyoxybenzylidene)hydrazinyl)-2-oxoethylthio)-4-oxobenzo*[g]*quinazolin-3*(4H)-*yl)benzenesulfonamide **(8)**

Yield, 89%; m.p. 253–255 °C. IR (KBr) cm^−1^: 3425 (s, OH stretch), 3372, 3291, 3167 (m, NH_2_, NH stretch), 3059 (s, CH arom. stretch), 2964, 2870 (s, CH aliph. stretch), 1676, 1664 (s, CO stretch), 1627 (m, CN stretch), 1389, 1161 (m, SO_2_ stretch). ^1^H NMR (DMSO-d_6_, 500 MHz)δ: 3.8 (s, 3H, OCH_3_), 4.3 (s, 2H, CH_2_), 6.8–8.2 (m, 13H, Ar-H), 8.8 (s, 1H, CH), 9.5 (s, 2H, SO_2_NH_2_), 11.5 (s, 1H, NH), 11.9 (s, 1H, OH). ^13^C NMR (DMSO-d_6_, 125.7 MHz)δ: 32.1, 56.0, 112.6, 117.0, 121.2, 123.7 (2), 124.1, 125.8, 126.6, 128.4 (2), 128.8 (2), 129.0, 129.1, 129.4, 131.3, 131.8, 133.7, 138.5, 142.8, 142.9, 148.4, 149.5, 161.7, 164.0, 175.1. MS *m/z* (%): 589 (M^+^) (33.41), 433 (100). Anal. Calcd. for C_28_H_23_N_5_O_6_S_2_ (589.64): C, 57.03; H, 3.93; N, 11.88. Found: C, 57.33; H, 4.30; N, 12.20.

#### 4-(2-(2-(2-(2,4-Dichlorobenzylidene)hydrazinyl)-2-oxoethylthio)-4-oxobenzo*[g]*-quinazolin-3*(4H)*-yl)benzenesulfonamide **(9)**

Yield, 79%; m.p. 235–237 °C. IR (KBr) cm^−1^: 3420, 3263, 3176 (m, NH_2,_ NH stretch), 3062 (s, CH arom. stretch), 2946, 2881 (s, CH aliph. stretch), 1692, 1681 (s, CO stretch), 1624 (m, CN stretch), 1392, 1157 (m, SO_2_ stretch), 742 (s, C-Cl stretch). ^1^H NMR (DMSO-d_6_, 500 MHz)δ: 4.4 (s, 2H, CH_2_), 7.3–8.5 (m, 13H, Ar-H), 8.8 (s, 1H, CH), 9.7 (s, 2H, SO_2_NH_2_), 11.9 (s, 1H, NH). ^13^C NMR (DMSO-d_6_, 125.7 MHz)δ: 31.2, 119.1, 119.8 (2), 123.6, 125.8, 125.9 (2), 126.8 (2), 127.1, 128.8, 129.6, 129.8, 131.0, 131.6, 131.8, 133.4, 133.7, 135.6, 135.7, 137.5, 139.6, 142.1, 159.4, 161.2, 176.7. MS *m/z* (%): 612 (M^+^) (25.13), 424 (100). Anal. Calcd. for C_27_H_19_Cl_2_N_5_O_4_S_2_ (612.51): C, 52.94; H, 3.13; N, 11.43. Found: C, 52.59; H, 3.02; N, 11.09.

#### 4-(2-(2-(2-(4-Bromobenzylidene)hydrazinyl)-2-oxoethylthio)-4-oxobenzo*[g]*quinazolin-3*(4H)*-yl)benzenesulfonamide **(10)**

Yield, 69%; m.p. 243–245 °C. IR (KBr) cm^−1^: 3456, 3360, 3218 (m, NH_2_, NH stretch), 3100 (s, CH arom. stretch), 2951, 2868 (s, CH aliph. stretch), 1698, 1687 (s, CO stretch), 1629 (m, CN stretch), 1394, 1159 (m, SO_2_ stretch). ^1^H NMR (DMSO-d_6_, 500 MHz)δ: 4.6 (s, 2H, CH_2_), 7.4–8.4 (m, 14H, Ar-H), 8.8 (s, 1H, CH), 9.8 (s, 2H, SO_2_NH_2_), 11.5 (s, 1H, NH). ^13^C NMR (DMSO-d_6_, 125.7 MHz)δ: 32.2, 119.0, 120.3 (2), 123.6, 124.8, 126.2 (2), 127.8 (2), 128.7 (2), 129.2, 130.6, 132.3, 132.5 (2), 132.5 (2). 133.1 (2), 134.0, 142.7, 143.6, 162.3, 163.9, 172.4. MS *m/z* (%): 622 (M^+^) (44.71), 395 (100). Anal. Calcd. for C_27_H_20_BrN_5_O_4_S_2_ (622.51): C, 52.09; H, 3.24; N, 11.25. Found: C, 52.38; H, 3.59; N, 11.55.

#### 4-(2-(2-(2-(Benzo*[d]*[1,3]dioxol-5-ylmethylene)hydrazinyl)-2-oxoethylthio)-4-oxobenzo*[g]*quinazolin-3*(4H)*-yl)benzenesulfonamide **(11)**

Yield, 77%; m.p. 259–261 °C. IR (KBr) cm^−1^: 3427, 3310, 3226 (m, NH_2,_ NH stretch), 3061 (s, CH arom. stretch), 2994, 2900 (s, CH aliph. stretch), 1690, 1681 (s, CO stretch), 1627 (m, CN stretch), 1346, 1155 (m, SO_2_ stretch). ^1^H NMR (DMSO-d_6_, 500 MHz)δ: 4.3 (s, 2H, CH_2_), 6.1 (s, 2H, O-CH_2_-O), 6.9–8.5 (m, 13H, Ar-H), 8.8 (s, 1H, CH), 9.9 (s, 2H, SO_2_NH_2_), 11.7 (s, 1H, NH). ^13^C NMR (DMSO-d_6_, 125.7 MHz)δ: 32.1, 102.0, 109.0, 114.6, 119.2, 120.6 (2), 120.9, 125.5, 125.9, 128.7 (2), 129.0 (2), 129.8 (2), 130.1, 130.5, 131.6, 131.9, 132.5, 134.8, 142.8 (2), 148.4, 149.8, 161.1, 173.6. MS *m/z* (%): 587 (M^+^) (53.73), 398 (100). Anal. Calcd. for C_28_H_21_N_5_O_6_S_2_ (587.63): C, 57.23; H, 3.60; N, 11.92. Found: C, 57.55; H, 3.92; N, 12.30.

#### *N,N-*Dimethyl-4-((2-(2-(4-oxo-3-(4-sulfamoylphenyl)-3,4-dihydrobenzo*[g]*quinazolin-2-ylthio)acetyl)hydrazono)methyl)-*1H*-imidazole-2-sulfonamide **(12)**

Yield, 83%; m.p. 238–240 °C. IR (KBr) cm^−1^: 3423, 3226, 3130 (m, NH_2,_ NH stretch), 3057 (s, CH arom. stretch), 2966, 2927 (s, CH aliph. stretch), 1690, 1681 (s, CO stretch), 1627 (m, CN stretch), 1392, 1176 (m, SO_2_ stretch). ^1^H NMR (DMSO-d_6_, 500 MHz)δ: 2.7 (s, 6H, N-(CH_3_)_2_), 4.5 (s, 2H, CH_2_), 7.3–8.4 (m, 10H, Ar-H), 8.8 (s, 1H, CH), 9.9 (s, 2H, SO_2_NH_2_), 11.6 (s, 1H, NH), 13.1 (s, 1H, NH imidazole). ^13^C NMR (DMSO-d_6_, 125.7 MHz)δ: 32.1, 38.3 (2), 117.0, 119.2, 120.3 (2), 121.9, 125.3, 126.8 (2), 126.9 (2), 127.4, 128.5, 129.0, 130.8, 132.6, 133.0, 134.8, 138.1, 138.8, 141.6, 161.8, 163.7, 172.0. MS *m/z* (%): 640 (M^+^) (6.51), 359 (100). Anal. Calcd. for C_26_H_24_N_8_O_6_S_3_ (640.71): C, 48.74; H, 3.78; N, 17.49. Found: C, 49.01; H, 4.09; N, 17.08.

#### 4-(2-(2-(2-Formylhydrazinyl)-2-oxoethylthio)-4-oxobenzo*[g]*quinazolin-3*(4H)*-yl)benzenesulfonamide **(13)**

A solution of **5** (4.55 g, 0.01 mol) in formic acid (10 ml) was refluxed for 8 h. The reaction mixture was concentrated, filtered and the solid obtained was crystallised from dioxane to give **13**. Yield, 84%; m.p. 167–169 °C. IR (KBr) cm^−1^: 3402, 3320, 3115 (m, NH_2,_ NH stretch), 3091 (s, CH arom. stretch), 2922, 2836 (s, CH aliph. stretch), 1695, 1681, 1658 (s, CO stretch), 1627 (m, CN stretch), 1369, 1155 (m, SO_2_ stretch). ^1^H NMR (DMSO-d_6_, 500 MHz)δ: 4.4 (s, 2H, CH_2_), 7.7–8.5 (m, 10H, Ar-H), 9.4 (s, 1H, NHCO), 10.2 (s, 2H, SO_2_NH_2_), 11.1 (s, 1H, NH). ^13^C NMR (DMSO-d_6_, 125.7 MHz)δ: 30.8, 120.0, 122.6 (2), 124.8, 126.6 (2), 127.8 (2), 127.9, 128.3, 130.1, 130.8, 133.7, 133.9, 135.0, 141.8, 159.5, 162.0, 167.3, 172.6. MS *m/z* (%): 483 (M^+^) (11.09), 168 (100). Anal. Calcd. for C_21_H_17_N_5_O_5_S_2_ (483.52): C, 52.16; H, 3.54; N, 14.48. Found: C, 52.50; H, 3.83; N, 14.77.

#### 4-(2-(2-(2-Acetylhydrazinyl)-2-oxoethylthio)-4-oxobenzo*[g]*quinazolin-3*(4H)*-yl)benzenesulfonamide **(14)**

A solution of **5** (4.55 g, 0.01 mol) in acetic acid (20 ml) was refluxed for 2 h. The reaction mixture was concentrated, filtered and the solid obtained was crystallised from acetic acid to give **14**. Yield, 89%; m.p. 201–203 °C. IR (KBr) cm^−1^: 3477, 3417, 3212 (m, NH_2,_ NH stretch), 3100 (s, CH arom. stretch), 2918, 2881 (s, CH aliph. stretch), 1734, 1710, 1680 (s, CO stretch), 1624 (m, CN stretch), 1371, 1166 (m, SO_2_ stretch). ^1^H NMR (DMSO-d_6_, 500 MHz)δ: 2.4 (s, 3H, COCH_3_), 4.5 (s, 2H, CH_2_), 7.3–8.8 (m, 10H, Ar-H), 10.1 (s, 2H, SO_2_NH_2_), 11.8, 13.2 (2 s, 2H, 2NH). ^13^C NMR (DMSO-d_6_, 125.7 MHz)δ: 24.9, 31.8, 118.0, 119.6 (2), 124.3, 124.6 (2), 126.1 (2), 126.7, 127.9, 128.8, 132.0, 133.0, 134.6, 135.8, 143.2, 159.6, 162.7, 170.1, 171.4. MS *m/z* (%): 497 (M^+^) (1.34), 341 (100). Anal. Calcd. for C_22_H_19_N_5_O_5_S_2_ (497.55): C, 53.11; H, 3.85; N, 14.08. Found: C, 53.44; H, 4.19; N, 14.38.

#### Ethyl *N*-2-(4-oxo-3-(4-sulfamoylphenyl)-3,4-dihydrobenzo*[g]*quinazolin-2ylthio)acetyl formohydrazonate **(15)**

A solution of **5** (4.55 g, 0.01 mol) in triethylorthoformate (15 ml) was refluxed for 7 h. The reaction mixture was concentrated under vacuum, filtered and the solid obtained was crystallised from ethanol to give **15**. Yield, 68%; m.p. 285–287 °C. IR (KBr) cm^−1^: 3427, 3310, 3271 (m, NH_2,_ NH stretch), 3100 (s, CH arom. stretch), 2981, 2935 (s, CH aliph. stretch), 1693, 1681 (s, CO stretch), 1627 (m, CN stretch), 1352, 1163 (m, SO_2_ stretch). ^1^H NMR (DMSO-d_6_, 500 MHz)δ: 1.3 (t, 3H, CH_3_ ethyl), 4.1 (q, 2H, CH_2_ ethyl), 4.5 (s, 2H, CH_2_), 7.5–8.5 (m, 10H, Ar-H), 8.8 (s, 1H, CH), 9.9 (s, 2H, SO_2_NH_2_), 11.5 (s, H, NH). ^13^C NMR (DMSO-d_6_, 125.7 MHz)δ: 15.6, 33.0, 62.8, 119.1, 119.8 (2), 124.8, 125.7 (2), 126.4, 126.8, 127.2, 127.7, 128.0, 131.2, 131.4, 133.0, 134.6, 140.8, 149.1, 158.6, 160.2, 172.5. MS *m/z* (%): 511 (M^+^) (5.29), 355 (100). Anal. Calcd. for C_23_H_21_N_5_O_5_S_2_ (511.57): C, 54.00; H, 4.14; N, 13.69. Found: C, 54.29; H, 4.39; N, 14.00.

#### 2-(2-(4-Oxo-3-(4-sulfamoylphenyl)-3,4-dihydrobenzo*[g]*quinazolin-2-ylthio)acetyl)-*N*-phenylhydrazinecarbothioamide **(16)**

A mixture of **5** (4.55 g, 0.01 mol) and phenyl isothiocyanate (1.35 g, 0.01 mol) in dioxane (20 ml) was refluxed for 12 h. The reaction mixture was poured onto ice water, filtered and the solid obtained was crystallised from ethanol to give **16**. Yield, 65%; m.p. 137–139 °C. IR (KBr) cm^−1^: 3310, 3207, 3120 (m, NH_2_, NH stretch), 3039 (s, CH arom. stretch), 2971, 2844 (s, CH aliph. stretch), 1688, 1654 (s, CO stretch), 1625 (m, CN stretch), 1346, 1163 (m, SO_2_ stretch), 1215 (s, CS stretch). ^1^H NMR (DMSO-d_6_, 500 MHz)δ: 4.6 (s, 2H, CH_2_), 7.0–8.4 (m, 15H, Ar-H), 9.8 (s, 2H, SO_2_NH_2_), 10.9 (s, 1H, NH), 13.9 (s, 1H, NH-C=S), 14.5 (s, 1H, NH-C=O). ^13^C NMR (DMSO-d_6_, 125.7 MHz)δ: 32.2, 119.7, 120.6 (2), 124.1, 124.8 (2), 125.0 (2), 126.3, 128.4 (2), 128.9 (2), 130.3 (3), 130.8 (2), 134.5 (2), 139.9 (3), 140.2, 142.6, 165.8 (3), 180.1. MS *m/z* (%): 601 (M^+^) (11.87), 74 (100). Anal. Calcd. For C_30_H_25_ClN_6_O_4_S (601.08): C, 59.95; H, 4.19; N, 13.98. Found: C, 60.23; H, 4.35; N, 14.12.

#### 4-(4-Oxo-2-(2-oxo-2-(2-oxoindolin-3-ylidene)hydrazinyl)ethylthio)benzo*[g]*quinazolin-3(4H)-yl) benzenesulfonamide **(17)**

To a solution of **5** (4.55 g, 0.01 mol) in DMF (15 ml), isatin (1.47 g, 0.01 mol) was added. The mixture was refluxed for 6 h, cooled, poured onto ice/water. The product formed was crystallised from dioxane to give **17**. Yield, 78%; m.p. 275–277 °C. IR (KBr) cm^−1^: 3441, 3221, 3160 (m, NH_2_, NH stretch), 3100 (s, CH arom. stretch), 2971, 2844 (s, CH aliph. stretch), 1708, 1695, 1680 (s, CO stretch), 1624 (m, CN stretch), 1398, 1159 (m, SO_2_ stretch). ^1^H NMR (DMSO-d_6_, 500 MHz)δ: 4.6 (s, 2H, CH_2_), 6.9–8.4 (m, 14H, Ar-H), 9.0 (s, 2H, SO_2_NH_2_), 11.0 (s, 1H, NH), 11.4 (s, 1H, NH isatin). ^13^C NMR (DMSO-d_6_, 125.7 MHz)δ: 32.6, 115.0, 119.0, 120.1, 120.8 (2), 122.6, 125.8, 126.7 (2), 126.9 (2), 127.1, 127.8, 127.9, 128.3, 130.9, 131.7, 131.8, 133.5, 133.7, 134.0, 140.8, 144.6, 159.8, 161.2, 165.5, 175.4. MS *m/z* (%): 584 (M^+^) (19.34), 428 (100). Anal. Calcd. for C_28_H_20_N_6_O_5_S_2_ (584.63): C, 57.52; H, 3.45; N, 14.38. Found: C, 57.18; H, 3.11; N, 14.13.

#### 4-(2-(2-(3-Methyl-5-oxo-4,5-dihydro-*1H*-pyrazol-1-yl)2-oxoethylthio)-4-oxobenzo*[g]*quinazolin-3*(4H)-*yl) benzenesulfonamide **(18)**

A mixture of **5** (4.55 g, 0.01 mol) and ethyl acetoacetate (1.30 g, 0.01 mol) in ethanol (30 ml) was refluxed for 8 h, cooled and the precipitate was filtered and crystallised from acetic acid to give **18**. Yield, 66%; m.p. 177–179 °C. IR (KBr) cm^−1^: 3425, 3191 (m, NH_2_ stretch), 3057 (s, CH arom. stretch), 2981, 2860 (s, CH aliph. stretch), 1705, 1695, 1660 (s, CO stretch), 1627 (m, CN stretch), 1398, 1163 (m, SO_2_ stretch). ^1^H NMR (DMSO-d_6_, 500 MHz)δ: 1.0 (s, 3H, CH_3_), 2.1 (s, 2H, CH_2_ pyrazole), 4.3 (s, 2H, CH_2_), 7.3–8.8 (m, 10H, Ar-H), 10.5 (s, 2H, SO_2_NH_2_). ^13^C NMR (DMSO-d_6_, 125.7 MHz)δ: 25.2, 31.1, 44.4, 119.0, 120.3 (2), 124.7, 124.9 (2), 126.8 (2), 128.4, 128.9, 131.2, 131.7, 133.0, 133.9, 136.0, 143.1, 158.3, 159.1, 161.7, 162.6, 170.8. MS *m/z* (%): 521 (M^+^) (0.86), 364 (100). Anal. Calcd. for C_24_H_19_N_5_O_5_S_2_ (521.57): C, 55.27; H, 3.67; N, 13.43. Found: C, 55.54; H, 3.95; N, 13.61.

#### 4-(2-(2-(3,5-Dimethyl-1H-pyrazol-1-yl)-2-oxoethylthio)-4-oxobenzo*[g]*quinazolin-3*(4H)-*yl) benzenesulfonamide **(19)**

A mixture of **5** (4.55 g, 0.01 mol) and acetylacetone (1.00 g, 0.01 mol) in ethanol (30 ml) was refluxed for 8 h. The precipitate formed after cooling was crystallised from ethanol to give **19**. Yield, 87%; m.p. 150–152 °C. IR (KBr) cm^−1^: 456, 3391 (m, NH_2_ stretch), 3055 (s, CH arom. stretch), 2961, 2866 (s, CH aliph. stretch), 1743, 1693 (s, CO stretch), 1624 (m, CN stretch), 1392, 1161 (m, SO_2_ stretch). ^1^H NMR (DMSO-d_6_, 500 MHz)δ: 2.4 (s, 6H, 2CH_3_), 4.3 (s, 2H, CH_2_), 6.1 (s, 1H, CH pyrazole), 7.4–8.6 (m, 10H, Ar-H), 9.9 (s, 2H, SO_2_NH_2_). ^13^C NMR (DMSO-d_6_, 125.7 MHz)δ: 13.9, 14.6, 19.0, 103.6, 118.6, 120.0 (2), 125.7, 125.9 (2), 126.1 (2), 126.4, 126.8, 128.2, 129.6, 131.2, 133.7, 135.6, 144.2, 144.4, 144.8, 161.0, 164.7, 203.1. MS *m/z* (%): 519 (M^+^) (2.26), 341 (100). Anal. Calcd. for C_25_H_21_N_5_O_4_S_2_ (519.60): C, 57.79; H, 4.07; N, 13.48. Found: C, 57.48; H, 3.90; N, 13.11.

#### 4-(2-(2-(3,5-Dioxopyrazolidin-1-yl)-2-oxoethylthio)-4-oxobenzo*[g]* quinazolin-3*(4H)-*yl) benzenesulfonamide **(20)**

To a solution of 0.5 g sodium in 20 ml ethanol, diethyl malonate (1.60 g, 0.01 mol) was added first and then the hydrazide **5** (4.55 g, 0.01 mol). The mixture was refluxed for 8 h, dissolved in water (30 ml), and filtered to remove the unreacted material, acidified with 10% HCl. The obtained solid was crystallised from dioxane to give **20**. Yield, 70%; m.p. 259–261 °C. IR (KBr) cm^−1^: 3421, 3331, 3215 (m, NH_2_, NH stretch), 3100 (s, CH arom. stretch), 2927, 2851 (s, CH aliph. stretch), 1712, 1698, 1680, 1668 (s, CO stretch), 1620 (m, CN stretch), 1348, 1155 (m, SO_2_ stretch). ^1^H NMR (DMSO-d_6_, 500 MHz)δ: 3.0 (s, 3H, CH_2_ pyrazole), 4.4 (s, 2H, CH_2_), 7.4–8.6 (m, 10H, Ar-H), 9.9 (s, 2H, SO_2_NH_2_), 11.8 (s, 1H, NH pyrazole). ^13^C NMR (DMSO-d_6_, 125.7 MHz)δ: 28.2, 48.4, 119.6, 121.4 (2), 124.1, 125.9 (2), 126.7 (2), 127.0, 129.8, 131.5, 131.8, 132.6, 134.6, 135.0, 143.3, 158.4, 162.1, 169.9 (2), 172.5. MS *m/z* (%): 523 (M^+^) (8.73), 353 (100). Anal. Calcd. for C_23_H_17_N_5_O_6_S_2_ (523.54): C, 52.76; H, 3.27; N, 13.38. Found: C, 52.98; H, 3.53; N, 13.55.

#### 4-(2-(2-(5-Amino-4-cyano-1H-pyrazol-1-yl)-2-oxoethylthio)-4-oxobenzo*[g]*quinazolin-3*(4H)-*yl) benzenesulfonamide **(21)**

A mixture of **5** (4.55 g, 0.01 mol) and 2-(ethoxymethylene)malononitrile (1.12 g, 0.01 mol) in methanol (30 ml) and glacial acetic acid (1 ml) was refluxed for 12 h. The solid obtained after cooling was crystallised from ethanol to give **21**. Yield, 90%; m.p. 250–252 °C. IR (KBr) cm^−1^: 3442, 3390, 3374 (m, NH_2_ stretch), 3078 (s, CH arom. stretch), 2931, 2861 (s, CH aliph. stretch), 2218 (m, CN stretch), 1695, 1681 (s, CO stretch), 1627 (m, CN stretch), 1350, 1161 (m, SO_2_ stretch). ^1^H NMR (DMSO-d_6_, 500 MHz)δ: 4.2 (s, 2H, CH_2_), 6.0 (s, 1H, CH pyrazole), 7.3–8.8 (m, 10H, Ar-H), 9.9 (s, 2H, SO_2_NH_2_), 10.4 (s, 2H, NH_2_). ^13^C NMR (DMSO-d_6_, 125.7 MHz)δ: 25.6, 78.9, 116.0, 121.1, 121.8 (2), 124.2, 125.6 (2), 126.1 (2), 126.8, 127.0, 128.3, 129.9, 131.0, 133.6, 135.8, 143.0, 143.7, 156.4, 159.1, 164.9, 202.7. MS *m/z* (%): 531 (M^+^) (1.88), 333 (100). Anal. Calcd. for C_24_H_17_N_7_O_4_S_2_ (531.57): C, 54.23; H, 3.22; N, 18.44. Found: C, 54.56; H, 3.55; N, 18.78.

#### Ethyl 5-amino-1-(2-(4-oxo-3-(4-sulfamoylphenyl)-3,4-dihydrobenzo*[g]*quinazolin-2-ylthio)acetyl)-*1H*-pyrazole-4-carboxylate **(22)**

A mixture of **5** (4.55 g, 0.01 mol) and ethyl 2-cyano-3-ethoxyacrylate (1.69 g, 0.01 mol) in methanol (30 ml) and glacial acetic acid (1 ml) was refluxed for 12 h. The solid obtained after cooling was crystallised from ethanol to give **22.** Yield, 77%; m.p. 248–250 °C. IR (KBr) cm^−1^: 3454, 3366, 3276 (m, NH_2_ stretch), 3057 (s, CH arom. stretch), 2981, 2843 (s, CH aliph. stretch), 1712, 1695, 1681 (s, CO stretch), 1627 (m, CN stretch), 1350, 1161 (m, SO_2_ stretch). ^1^H NMR (DMSO-d_6_, 500 MHz)δ: 1.2 (t, 3H, CH_3_ ester), 4.2 (s, 2H, CH_2_), 4.4 (q, 2H, CH_2_ ester), 6.2 (s, 2H, NH_2_), 7.3–8.6 (m, 10H, Ar-H), 8.7 (s, 1H, CH pyrazole), 9.9 (s, 2H, SO_2_NH_2_). ^13^C NMR (DMSO-d_6_, 125.7 MHz)δ: 17.4, 23.8, 58.7, 104.3, 121.2, 121.8 (2), 125.0, 125.6 (2), 125.9 (2), 127.1, 128.8, 128.9, 134.2, 134.4, 134.6, 134.9, 135.8, 146.1, 150.2, 164.1, 168.0, 169.7, 203.6. MS *m/z* (%): 578 (M^+^) (77.09), 328 (100). Anal. Calcd. for C_26_H_22_N_6_O_6_S_2_ (578.62): C, 53.97; H, 3.83; N, 14.52. Found: C, 53.62; H, 3.55; N, 14.25.

#### 4-(2-(2-(5-Amino-4-cyano-3-methyl-*1H*-pyrazol-1-yl)-2-oxoethylthio)-4-oxobenzo*[g]* quinazo-lin-3*(4H)-*yl) benzenesulfonamide **(23)**

A mixture of **5** (4.55 g, 0.01 mol) and 2-(1-ethoxyethylidine))malononitrile (1.36 g, 0.01 mol) in methanol (30 ml) and glacial acetic acid (1 ml) was refluxed for 12 h. The solid obtained after cooling was crystallised from ethanol to give **23.** Yield, 87%; m.p. 255–257 °C. IR (KBr) cm^−1^: 3421, 3319, 3280 (m, NH_2_ stretch), 3053 (s, CH arom. stretch), 2913, 2891 (s, CH aliph. stretch), 2212 (m, CN stretch), 1690, 1681 (s, CO stretch), 1625 (m, CN stretch), 1350, 1159 (m, SO_2_ stretch). ^1^H NMR (DMSO-d_6_, 500 MHz)δ: 2.6 (s, 3H, CH_3_), 4.2 (s, 2H, CH_2_), 6.2 (s, 2H, NH_2_), 7.4–8.7 (m, 10H, Ar-H), 10.8 (s, 2H, SO_2_NH_2_). ^13^C NMR (DMSO-d_6_, 125.7 MHz)δ: 12.4, 22.8, 78.1, 116.8, 122.3, 122.8 (2), 127.7, 127.9 (2), 128.0 (2), 128.5, 128.9, 129.6, 131.0, 131.8, 133.2, 136.0, 148.2 (2), 158.1, 161.4, 168.9, 198.7. MS *m/z* (%): 545 (M^+^) (23.81), 390 (100). Anal. Calcd. for C_25_H_19_N_7_O_4_S_2_ (545.59): C, 55.04; H, 3.51; N, 17.97. Found: C, 55.32; H, 3.84; N, 18.21.

#### Ethyl 5-amino-3-methyl-1-(2-(4-oxo-3-(4-sulfamoylphenyl)-3, 4-dihydrobenzo*[g]*quinazolin-2-ylthio)acetyl)-*1H*-pyrazole-4-carboxylate **(24)**

A mixture of **5** (4.55 g, 0.01 mol) and ethyl 2-cyano-3-ethoxybut-2-enoate (1.83 g, 0.01 mol) in methanol (30 ml) and glacial acetic acid (1 ml) was refluxed for 12 h. The solid obtained after cooling was crystallised from ethanol to give **24.** Yield, 58%; m.p. 248–250 °C. IR (KBr) cm^−1^: 3444, 3312, 3266 (m, NH_2_ stretch), 3100 (s, CH arom. stretch), 2955, 2871 (s, CH aliph. stretch), 1741, 1694, 1681 (s, CO stretch), 1627 (m, CN stretch), 1346, 1159 (m, SO_2_ stretch). ^1^H NMR (DMSO-d_6_, 500 MHz)δ: 1.2 (t, 3H, CH_3_ ester), 2.6 (s, 3H, CH3 pyrazole), 4.2 (s, 2H, CH_2_), 4.3 (q, 2H, CH_2_ ester), 6.0 (s, 2H, NH_2_), 7.3–8.6 (m, 10H, Ar-H), 9.9 (s, 2H, SO_2_NH_2_). ^13^C NMR (DMSO-d_6_, 125.7 MHz)δ: 12.2, 14.1, 23.8, 62.6, 109.4, 121.2, 121.8 (2), 125.2, 126.7 (2), 128.3 (2), 128.4, 128.6, 129.3, 135.2, 137.1, 137.4, 137.9, 139.2, 148.7, 158.0, 162.6, 168.7, 169.1, 205.6. MS *m/z* (%): 592 (M^+^) (61.23), 381 (100). Anal. Calcd. for C_27_H_24_N_6_O_6_S_2_ (592.65): C, 54.72; H, 4.08; N, 14.18. Found: C, 55.02; H, 4.29; N, 14.47.

## Biological evaluation

### MTT cytotoxicity assay

MCF-7 BC cells and MCF-12 A normal breast cells (obtained from VACSERA, Cairo, Egypt) were obtained from American Type Culture Collection (Manassas, VA), cells were cultured using Dulbecco's Modified Eagle's Medium (DMEM) (Invitrogen/Life Technologies MA, USA) supplemented with 10% fetal bovine serum (Hyclone USA), 10 µg/ml of insulin (Sigma, St. Louis, MO), and 1% penicillin-streptomycin. Plate cells (cells density 1.2–1.8 × 10,000 cells/well) in a volume of 100 µl complete growth medium +100 ul of the tested compound per well in a 96-well plate for 24 h before the MTT assay. Then, briefly rinse the cell layer with 0.25% (w/v) Trypsin 0.53 mm ethylenediamine tetraacetic acid (EDTA) solution to remove all traces of serum which contains trypsin inhibitor. Incubate cultures at 37 °C for 24 h. Add reconstituted MTT in an amount equal to 10% of the culture medium volume. Incubate for 2–4 h. Measure absorbance at a wavelength of 570 nm. The IC_50_ values were calculated according to the equation for Boltzmann sigmoidal concentration-response curve using the nonlinear regression fitting models (Graph Pad, Prism version 5, La Jolla, CA).

### VEGFR-2 assay

VEGFR-2 activity and IC_50_ of the selected compounds were determined in MCF-7 cells. Cells were cultured using the above-mentioned methods. The kinase activity of VEGFR-2 was measured by the use of a phosphotyrosine antibody with the Alpha Screen system (PerkinElmer) according to manufacturer’s instructions. The tested compounds at final concentrations ranging from 0 to 100 mg/ml and enzyme were incubated for 5 min at room temperature. The reactions were quenched by the addition of 25 ml of 100 mm EDTA. The plate was incubated in the dark overnight and then read by ELISA Reader (PerkinElmer). Percent inhibition was calculated by the comparison of compounds treated to control incubations, and the data were compared with Vandetanib as a standard VEGFR-2 inhibitor.

### Effect on active caspase-3

To determine the effect of the synthesised compounds on apoptosis, the active caspase-3 level was measured by using Quantikine-Human active Caspase-3 Immunoassay (R&D Systems, Inc. Minneapolis, MN) according to the manufacturer protocol. Cells were obtained from American Type Culture Collection, then were grown in Roswell Park Memorial Institute (RPMI) 1640 containing 10% foetal bovine serum at 37 °C, stimulated with the compounds to be tested for caspase3, and lysed with Cell Extraction Buffer. This lysate was diluted in Standard Diluent Buffer over the range of the assay, the optical density of each well was determined within 30 min using a microplate reader set at 450 nm to determine the human active caspase-3 content.

### Effect on BAX and bcl-2 levels

Cells were grown in RPMI 1640 containing 10% foetal bovine serum at 37 °C, stimulated with the compounds to be tested for Bax, and lysed with cell extraction buffer. This lysate was diluted in the standard diluent buffer over the range of the assay and measured for human active Bax and BCl2 content according to the reported method^29^.

### Analysis of cell cycle distribution

To determine the effect of compounds **9** and vandetanib on the cell cycle distribution MCF-7 cell line; cell cycle analysis was performed using the CycleTEST™ PLUS DNA Reagent Kit (Becton Dickinson Immunocytometry Systems, San Jose, CA). Control cells with known DNA content (PBMCs) were used as a reference point for determining the DI (DNA Index) for the test samples. The cells were stained with propidium iodide stain following the procedure provided by the kit and then run on the DNA cytometer. Cell cycle distribution was calculated using CELLQUEST software (Becton Dickinson Immunocytometry Systems).

## Molecular docking

All the molecular modelling studies were carried out using MOE, 10.2008 software (North Buona, Singapore). Energy minimisations were performed with a root mean standard deviation (RMSD) gradient of 0.05 kcal mol^−1 ^Å^−1^ with an MMFF94X force field and the partial charges were calculated. The protein data bank file (PDB: 3U6J) was selected for this purpose. The file contains VEGFR-2 co-crystallised with a *N*-(4-((6,7-dimethoxyquinolin-4-yl)oxy)-3-fluorophenyl)1,5-dimethyl-3-oxo-2-phenyl-2,3-dihydro-1H-pyrazole-4-carboxamide, obtained from protein data bank. The enzyme was prepared for docking studies: (i) Water molecules were ignored; (ii) hydrogen atoms were added to the enzyme; (iii) MOE Alpha Site Finder was used for the active sites search in the enzyme and (iv) removal of the co-crystallised ligand and docking of the new targeted structures.

## Results and discussion

### Chemistry

**Scheme 1.**
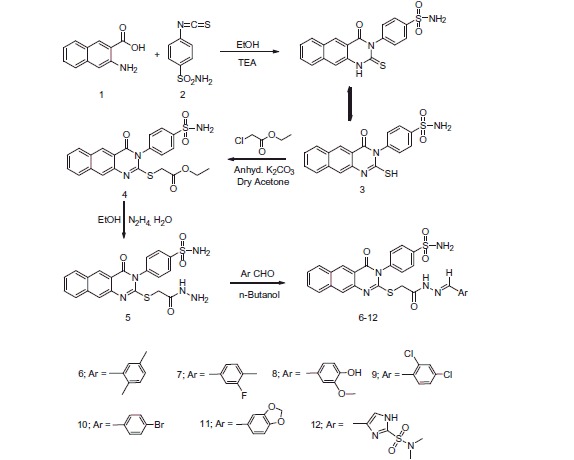
Formation of benzoquinazoline-sulfonamide derivatives **3–12**.

**Scheme 2.**
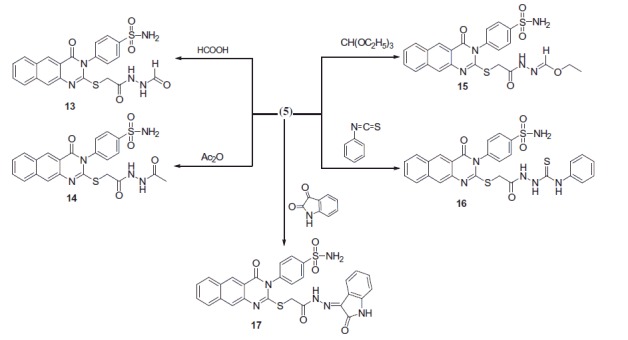
Formation of benzoquinazoline-sulfonamide derivatives **13–19**.

**Scheme 3.**
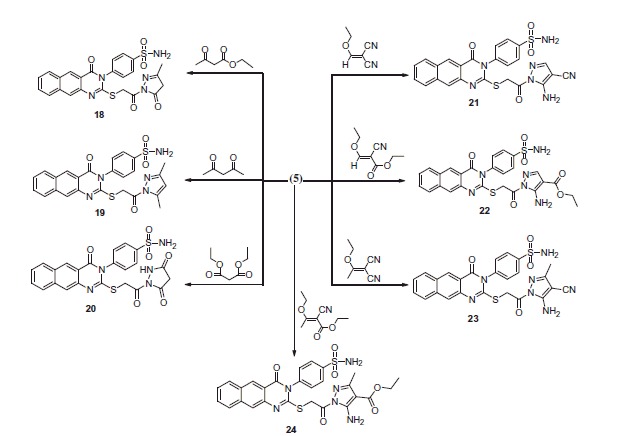
Formation of benzoquinazoline-sulfonamide derivatives **18–24**.

Schemes 1–3 report the synthetic pathways utilised to obtain the target compounds (**5–24**), from the reaction of 3-amino-2-naphthoic acid **1** with 4-isothiocyanatobenzenesulfonamide **2**^28^ in ethanol containing triethylamine to yield the 4-(2-mercapto-4-oxobenzo[*g*]quinazolin-3(4*H*)-yl) benzenesulfonamide **3.**^1^H-NMR of **3** revealed a singlet at δ 13.2 attributed to the SH. ^13^C-NMR exhibited two signals at δ 160.3 and 176.0 attributed to (C-SH) and (CO). The reaction of **3** with ethyl chloroacetate in dry acetone and anhydrous K_2_CO_3_ gave the ethyl-2-(4-oxo-3-(4-sulfamoylphenyl)-3,4-dihydrobenzo[*g*]quinazolin-2-ylthio)acetate **4**, (Scheme 1). IR of **4** revealed bands at 1749, 1691 cm^−1^ for the introduced CO groups. ^1^H-NMR displayed triplet at δ 1.2 attributed to CH_3_, singlet at δ 4.0 for the CH_2_ and quartet at δ 4.3 for the CH_2_ ester. ^13^C-NMR exhibited new up-field signals at δ 14.4, 34.9, 61.3 for the CH_3_, CH_2_, CH_2_ ester and another signal at δ 168.8 due to the CO ester. The reaction of **4** with hydrazine hydrate in ethanol yielded the 4-(2-(2-hydrazinyl-2-oxoethylthio)-4-oxobenzo[*g*]quinazolin-3(4H)-yl) benzenesulfonamide **5**, (Scheme 1). ^1^H-NMR of **5** displayed two singlets at δ 4.5 and 9.7 corresponding to NH_2_ and NH. The reaction of **5** with a series of aromatic aldehydes yielded the corresponding hydrazinyl derivatives **6–12** (Scheme 1). ^1^H-NMR of **6–12** displayed a singlet corresponding to the CH. While, ^13^C-NMR exhibited a new signal corresponding to the CH, at their specified regions.

Reaction of **5** with formic acid and/or acetic anhydride gave the 4-(2-(2-(2-formylhydrazinyl)-2-oxoethylthio)-4-oxobenzo[g]quinazolin-3(4H)-yl) benzenesulfonamide **13** and 4-(2-(2-(2-Acetylhydrazinyl)-2-oxoethylthio)-4-oxobenzo[g] quinazolin-3(4H)-yl)benzenesulfonamide **14** (Scheme 2). ^1^H-NMR of **13** displayed a singlet at δ 9.4 corresponding to the NHCO group. ^13^C-NMR of **13** exhibited a signal at δ 167.3 assigned to the introduced CO. ^1^H-NMR of **14** displayed an up-field singlet at δ 2.4 for the CH_3_ protons. ^13^C-NMR exhibited two new signals at δ 24.9 and 170.1 for the CH_3_ and CO groups, respectively. The reaction of **5** with triethyl orthoformate yielded the ethyl-*N*-2-(4-oxo-3-(4-sulfamoylphenyl)-3,4-dihydrobenzo[g]quinazolin-2ylthio)acetylformohydrazonate **15**, (Scheme 2). ^1^H-NMR of **15** displayed triplet at δ 1.3 attributed to the CH_3_, quartet at δ 4.1 for the CH_2_ and singlet at δ 8.8 for the CH. ^13^C-NMR exhibited new signals at δ 15.6, 62.8 and 149.1 for the CH_3_, CH_2_ and CH groups. The reaction of **5** with phenyl isothiocyanate in dioxane yielded the 2-(2-(4-oxo-3-(4-sulfamoylphenyl)-3,4-dihydrobenzo[*g*]quinazolin-2-ylthio)acetyl)-*N*-phenylhydrazine carbothioamide **16** (Scheme 2). ^1^H-NMR of **16** displayed a singlet at δ 13.9 for the (NH–C=S) and another singlet at δ 14.5 attributed to (NH–C=O). ^13^C-NMR exhibited a signal at δ 180.1 for the (C=S). Reaction of **5** with isatin in dimethylformamide yielded the 4-(4-oxo-2-(2-oxo-2-(2-oxoindolin-3-ylidene)hydrazinyl)ethylthio)benzo[*g*]quinazolin-3(4*H*)-yl) benzenesulfonamide **17** (Scheme 2). ^1^H-NMR of **17** displayed a singlet at δ 11.4 for the introduced NH. The reaction of **5** with ethyl acetoacetate and/or acetylacetone yielded the corresponding 4-(2-(2-(3-methyl-5-oxo-4,5-dihydro-*1H*-pyrazol-1-yl)-2-oxoethylthio)-4-oxobenzo[*g*]-quinazolin-3(4*H*)-yl) benzenesulfonamide **18** and 4-(2-(2-(3,5-dimethyl-1*H*-pyrazol-1-yl)-2-oxoethylthio)-4-oxobenzo[*g*]quinazolin-3(4*H*)-yl)benzenesulfonamide **19** (Scheme 2). The reaction proceeded according to similarly reported reactions^30^.

The reaction of **5** with a series of diethyl malonate, 2-(ethoxymethylene) malononitrile, ethyl 2-cyano-3-ethoxyacrylate, 2-(1-ethoxyethylidine) malononitrile and ethyl 2-cyano-3-ethoxybut-2-enoate yielded the corresponding pyrazole derivatives **20–24** (Scheme 3). The structures were confirmed on the basis of elemental analyses and spectroscopic data.

## Biological evaluation

### *In vitro* cytotoxic activity against MCF-7

The newly synthesised compounds **5–24** were screened for their *in vitro* cytotoxic activity against MCF-7 cell line. From the results in Table 1, it is obvious that compounds **5–24** cytotoxicity ranges from 0.1 to 26.65 µm. The hydrazinyl derivatives **5–17** IC_50_ ranges from 0.1 to 26.65 µm, while the pyrazolyl derivatives **18–24** from 0.11 to 1.37 µm, which may give an indication that the pyrazole ring is important for anticancer activity. Compounds **9**, **20, 22** and **23** (IC_50_= 0.10–0.17 µm) are the most potent in this study and possess better cytotoxic activity than the reference drug vandetanib (IC_50_= 0.24 µm). The 2,4-dichlorobenzylidene hydrazinyl derivative **9** has proven to be the most potent, followed by the 4-carboxylate **22**, the 4-cyanopyrazolyl **23** and the 3-oxopyrazolyl derivative **20**. Compounds **5–7, 10–14, 17–19, 21** and **24** showed moderate cytotoxic activity (IC_50_ 0.38–6.40 µm), while compounds **8, 15** and **16** showed relatively poor cytotoxic activity (IC_50_ 12.37–26.65 µm).

**Table 1.** VEGFR-2 inhibitory activity and anti-proliferative activity against MCF-7 cell line.

Cpd no.	IC_50_ against MCF-7 (μm)	% Inhibition of VEGFR-2 enzyme	IC_50_ on VEGFR-2 enzyme (μm)
**5**	0.38	68.44	NT
**6**	0.59	69.95	NT
**7**	0.63	72.19	NT
**8**	26.65	54.35	NT
**9**	0.10	88.09	0.64
**10**	0.68	64.89	NT
**11**	0.58	71.29	NT
**12**	0.86	49.60	NT
**13**	6.40	10.45	NT
**14**	0.78	62.69	NT
**15**	12.37	48.06	NT
**16**	13.31	38.16	NT
**17**	0.72	60.68	NT
**18**	0.66	64.12	NT
**19**	1.12	47.60	NT
**20**	0.17	85.69	0.86
**21**	1.30	55.34	NT
**22**	0.11	84.54	0.83
**23**	0.16	86.25	1.04
**24**	1.37	34.68	NT
Vandetanib	0.24	84.72	1.01

NT: not tested.

### VEGFR-2 inhibition

The VEGFR-2 inhibitory activity of the targeted compounds **5–24** was investigated. Results showed that most of the tested compounds have high inhibitory activity ranging from 88.09% to 34.68%, except for compound **13** that showed poor inhibitory profile 10.45%. Compounds **9**, **20, 22** and **23** showed the highest inhibition percentages ranging from 88.09% to 84.54%. The IC_50_ values for those four compounds were also recorded as 0.64, 0.86, 0.84 and 1.04 µm, respectively (Table 1). The 2,4-dichlorobenzylidene hydrazinyl derivative **9** showed the lowest IC_50_ value (0.64 µm), and 88.09% VEGFR-2 inhibition. Vandetanib was used as a reference drug with IC_50_ 1.01 µm, and 84.72% VEGFR-2 inhibition.

### Apoptosis studies

From the above results, most of the synthesised compounds proved to have VEGFR-2 inhibitory activity. As mentioned before, the inhibition of VEGFR-2 enzyme can lead to the induction of apoptosis^26^^,^^27^. Therefore, the ability of the most potent compound **9** to induce the apoptosis cascade will be investigated.

### Activation of caspase-3

**Figure 3.**
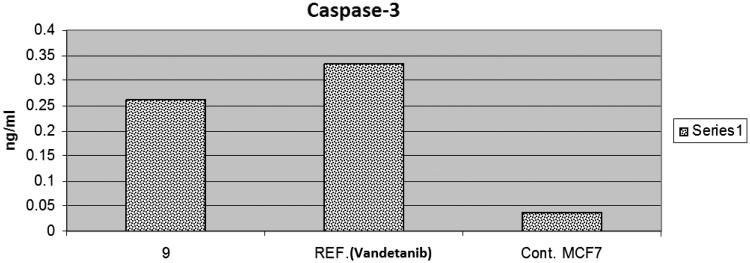
The effect of compound **9** and vandetanib on the activation of caspase-3 in MCF-7 cells.

Activation of caspases plays a key role in the initiation and execution of the apoptotic process^31^. Caspase 3 is initiated by the death cascade. It is activated by the upstream of caspase 8 and 9. So, it acts as a convergence point in different signalling pathways^32^^,^^33^. The effect of compound **9** on caspase 3 was evaluated and compared to vandetanib as a reference drug. It showed an increase in the level of active caspase 3 by 7-folds, compared to the control cells. While vandetanib induced caspase 3 approximately by 10 times (Table 2, Figure 3).

**Table 2.** Effect of compound **9** on the active caspases-3 in MCF-7 cells.

Cpd no.	Caspase-3 ng/ml
**9**	0.262
Vandetanib	0.334
Control	0.037

### Effects on bcl-2 family proteins

**Figure 4.**
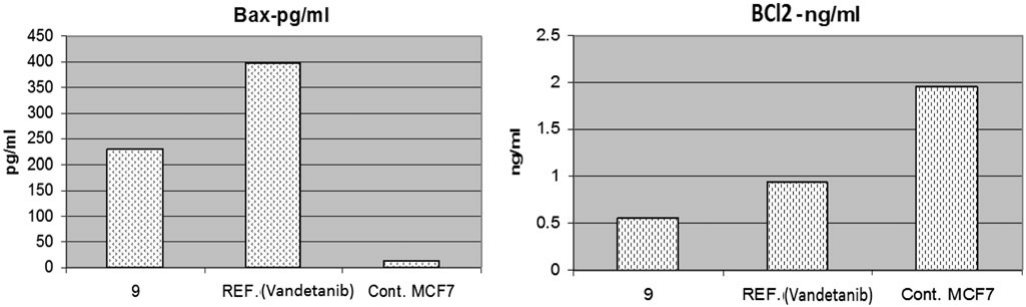
The effect of compound **9** and vandetanib on the level of Bax and BCl2.

The B-cell lymphoma protein 2 (Bcl-2) family plays a key role in tumour progression or inhibition of intrinsic apoptotic pathway triggered by mitochondrial dysfunction^34^. The Bcl2 protein inhibits apoptosis (anti-apoptotic) while Bax stimulates it (proapoptotic). Thus, the balance between these two different opposing proteins regulates the cell fate^35^^,^^36^. Increments in the Bax/Bcl2 ratio trigger the release of mitochondrial cytochrome C into the cytosol which in turn potentiates a cascade of caspases that ultimately leads to activation of caspase 3; the apoptosis executioner^37^. In this study, MCF-7 cells were treated with the IC_50_ of compound **9** and their effect on the expression levels of Bcl2, and Bax were determined as illustrated in Table 3 and Figure 4.

**Table 3.** Effect of compound **9** on the expression of the gene of some apoptosis key markers.

Cpd no.	BAX (pg/ml)	BCl2 (ng/ml)
**9**	230.8	0.561
Vandetanib	397.9	0.948
Control	13.68	1.953

As shown by the results, compound **9** boosted the level of the proapoptotic protein; Bax to 230.8 pg/ml compared to the control (13.68 pg/ml). Moreover, compound **9** markedly reduced the levels of the anti-apoptotic proteins Bcl2 to 0.561 ng/ml compared to the control (1.953 ng/ml). While vandetanib boosted the level of Bax to 397.9 pg/ml and reduced the level of the anti-apoptotic proteins Bcl2 to 0.948 ng/ml. Collectively, these findings proved that both compound **9** and vandetanib markedly increased Bax level and downregulated Bcl2 proved undoubtedly their proapoptotic effect.

### Cell cycle analysis

**Figure 5.**
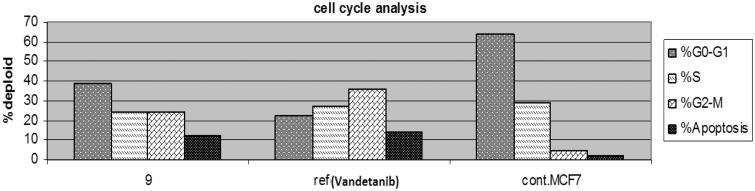
The effect of compound **9** and vandetanib on the cell cycle phases.

**Figure 6.**
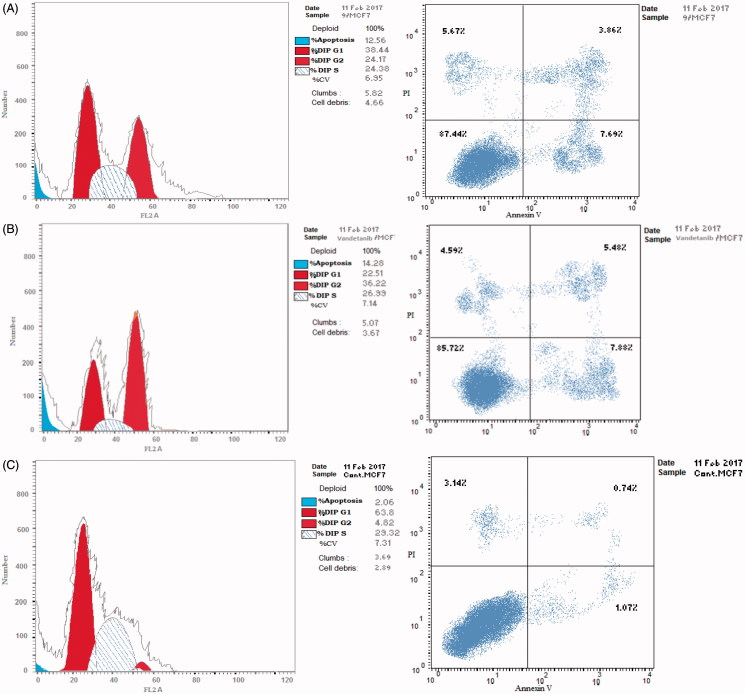
The effect on the phases of the cell cycle (A) compound **9**, (B) Vandetanib, (C) control MCF-7 cells.

In general, the anticancer agents abort the growth and proliferation of cancerous cells by arresting cell division at various checkpoints. These checkpoints present at G1/S phase, S-phase and G2/M phases^38^. Treatment of the cancer cells with anticancer agents can determine at which phase apoptosis occurs in the cell cycle. In the current study, MCF-7 cells were treated with compound **9** at its IC_50_ (0.10 µm). The obtained data (Figures 5 and 6(A)) obviously indicate that compound **9** arrested the cell cycle at a G2/M phase when compared to the untreated control (24.17% and 4.82%, respectively) (Figure 6(C)). While vandetanib arrested the cell cycle at the G2/M phase by 36.22% (Figure 6(B)). Parallel to these findings, the cell population in G1 and S phases decrease after treatment (38.44 and 24.38% versus 22.51 and 29.32%, respectively) in the case of compound **9**. While in the case of vandetanib, the cell population in G1 and S phases markedly decreases after treatment to (22.51% and 26.32%, respectively). These results reveal that in MCF-7 cells, cell cycle arrest occurs in the G2/M phase in case of compound **9** and vandetanib.

### Cytotoxicity test

**Figure 7.**
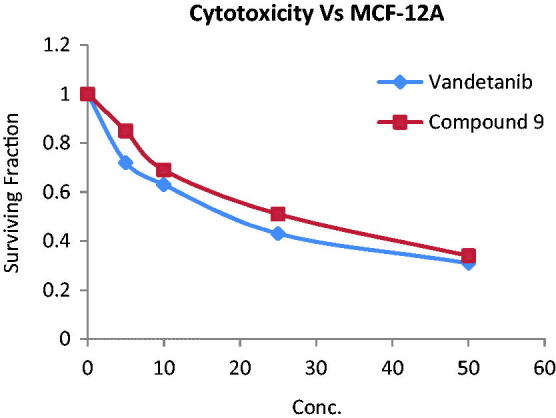
Cytotoxic activity of compound **9** and vandetanib towards MCF-12 A normal breast cell line.

The cytotoxic effects of compound **9** and vandetanib were tested on normal breast cells MCF-12 A using sulforhodamine B assay^39^. Both compounds showed mild cytotoxic effect with an IC_50_ of 29.41 and 22.06 µm, respectively (Figure 7). This result indicates the selectivity of compounds **9** and vandetanib for tumour BC cells and their relative safety for normal breast cells.

## Molecular docking

**Figure 8.**
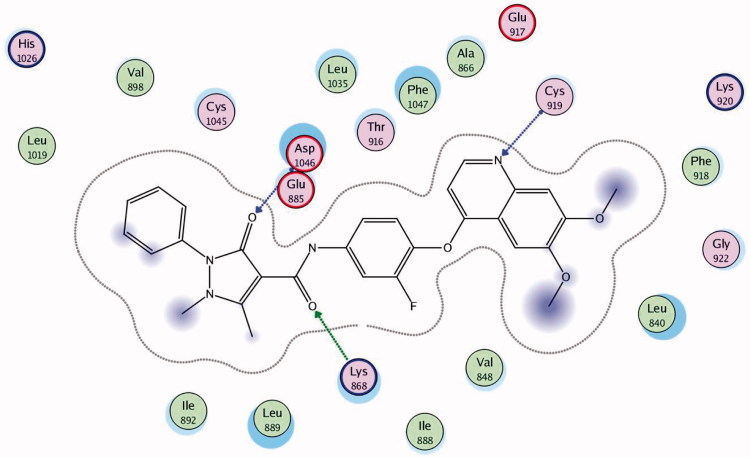
2D interaction of the co-crystallised ligand inside the active site of **3U6J**.

Docking was performed with the VEGFR-2 crystal structure (PDB code: 3U6J)^23^ by MOE 2008.10. The file contains the enzyme co-crystallised with *N*-(4-((6,7-dimethoxyquinolin-4-yl)oxy)-3-fluorophenyl)1,5-dimethyl-3-oxo-2-phenyl-2,3-dihydro-1H-pyrazole-4-carboxamide. Docking protocol was verified by re-docking of the co-crystallised ligand in the vicinity of the active site of the enzyme with energy score (S) = −10.82 kcal/mol and RMSD = 0.49 (Figure 8). The quinoline ligand interacts with the active site of 3U6J by three hydrogen bonds, Asp 1046 with carbonyl of the pyrazole by a hydrogen bond of 2.67 A°, Lys 868 with the carbonyl of the amide group by a hydrogen bond of 3.05 A°, Cys 919 with the nitrogen of quinoline by a hydrogen bond of 2.81 A°, and the phenyl ring is deeply immersed inside the hydrophobic pocket containing Ile888, Ile892, Leu1019 and Ile1049.

**Figure 9.**
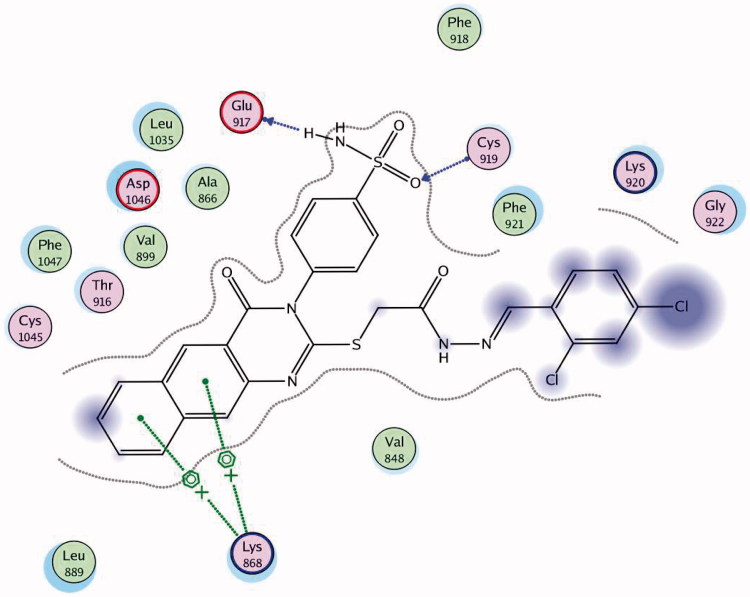
2D interaction of compound **9** inside the active site of **3U6J**.

**Figure 10.**
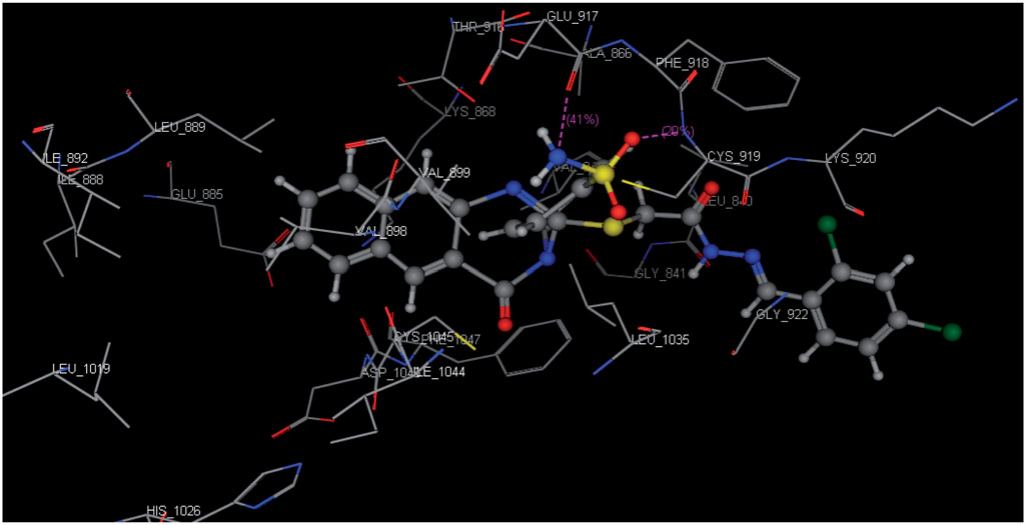
3D interaction of compound **9** inside the binding site of **3U6J**.

**Figure 11.**
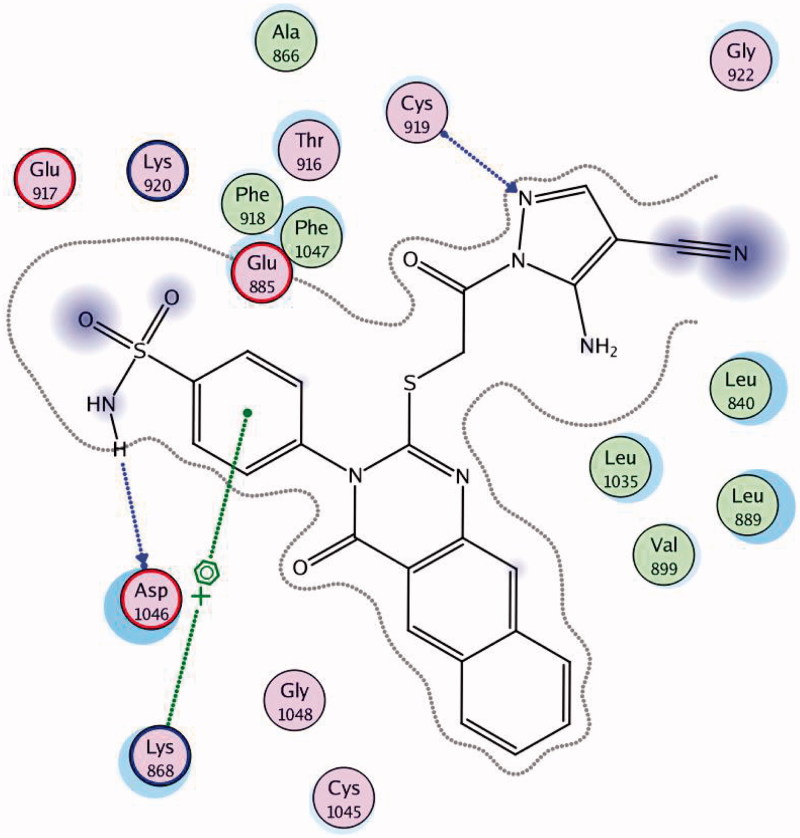
2D interaction of compound **22** inside the active site of **3U6J**.

**Figure 12.**
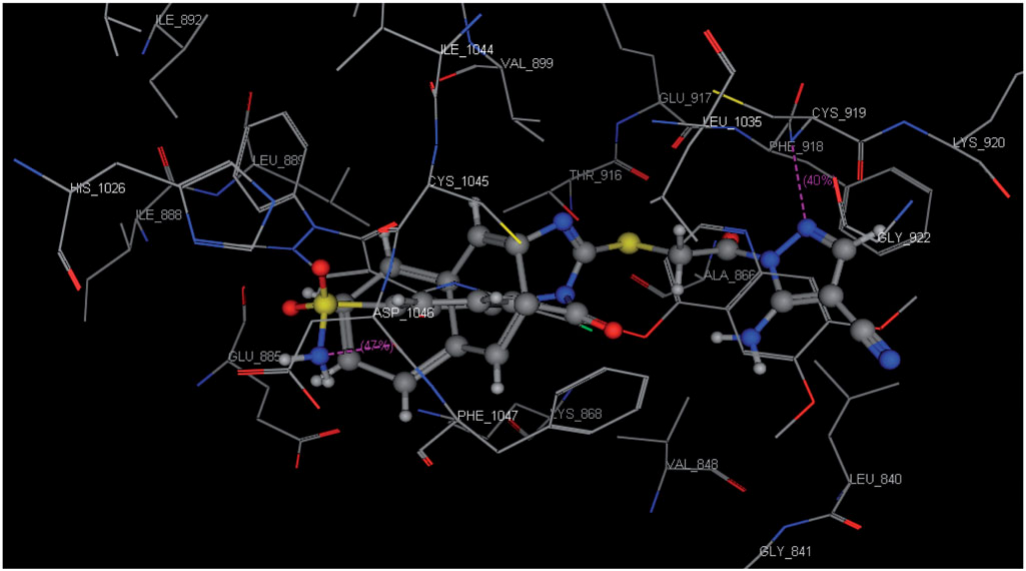
3D interaction of compound **22** inside the binding site of **3U6J**.

Docking was performed for all the synthesised compounds. Energy scores (S), as well as amino acids interactions of compounds **9** and **22**, were listed in Table 4. The best docking score was achieved by compound **9** with a value = −12.16 Kcal/mol. Compound **9** interacted with Cys 919 by a hydrogen bond of 2.54 A°, Glu 885 with a hydrogen bond of 2.89 A° and Lys 868 by cation-pi interaction with the two phenyl rings of Benzo[*g*]quinazolin (Figures 9 and 10). While compound **22** interacted with Asp 1046 by a hydrogen bond of 2.56 A°, Cys 919 by a hydrogen bond of 2.85 A° and Lys 868 by cation-pi interaction with the phenyl ring of sulfonamide (S = −10.95 Kcal/mol) (Figures 11 and 12).

**Table 4.** Docking results of the promising compounds inside the **3U6J** active site.

Compound	Energy score (S) (kcal/mol)	Amino acids	Interacting groups	Length (Å)
Ligand	−10.82	Asp 1046	CO of pyrazole	2.21
		Lys 868	CO of amide	2.92
		Cys 919	N of quinoline	3.10
**9**	−12.16	Lys 868	Benzo[*g*]quinazolin	4.12
		Cys 919	SO_2_ of sulfonamide	2.54
		Glu 917	NH of sulfonamide	2.89
**22**	−10.95	Asp 1046	NH of sulfonamide	2.56
		Lys 868	Ph of sulfonamide	3.02
		Cys 919	N of pyrazole	2.85

## Conclusions

In summary, we had synthesised a novel series of benzo[*g*]quinazolin bearing benzenesulfonamide moiety. All the synthesised compounds were screened for their cytotoxic activity against MCF-7 BC cell line, and their percentage inhibition against the VEGFR-2 enzyme. Compounds **9, 20, 22** and **23** have proved to be the most cytotoxic (IC_50_ ranging from 0.10 to 0.17 µm) and exhibited the highest inhibitory profile against VEGFR-2 (% inhibition ranging from 88.09 to 84.54%). These four compounds were found to potently inhibit VEGFR-2 at IC_50_ values of 0.64, 0.86, 0.83 and 1.04 µm, respectively. The 2,4-dichlorobenzylidene hydrazinyl derivative **9**, the most potent, was further selected to investigate its apoptotic inducing effect. Compound **9** showed an increase in the level of active caspase 3 by 7-folds, an upregulation in BAX and downregulation in Bcl2 levels, compared to the control cells and vandetanib. Cell cycle analysis of compound **9** showed that it arrested the cell population at G2/M phase. These findings have proven that compound **9** and vandetanib are apoptosis inducers. Both compound **9** and vandetanib were screened for their cytotoxicity against normal breast cell line (MCF-12 A) and were found to possess mild activity. Finally, molecular modelling studies were carried out, which showed that the benzo[*g*]quinazolin derivatives **5–24** bind to the target enzyme in the same pattern as the co-crystallised ligand.

## Supplementary Material

IENZ_1334650_Supplementary_Material.pdf
