# Supplementary material for: VEGFR-2 inhibitors and apoptosis inducers: synthesis and molecular design of new benzo[g]quinazolin bearing benzenesulfonamide moiety
Source: J Enzyme Inhib Med Chem. 2017 Jun 29;32(1):893–907. doi: 10.1080/14756366.2017.1334650 (PMC6445170; doi:10.1080/14756366.2017.1334650)
Supplement: IENZ_1334650_Supplementary_Material.pdf [file IENZ_A_1334650_SM4483.pdf]

SHIMADZU

KSU - College of Pharmacy
Medicinal, Aromatic & Poisnous Plants Research Centre
FT- IR Report

Sample Name: Ester

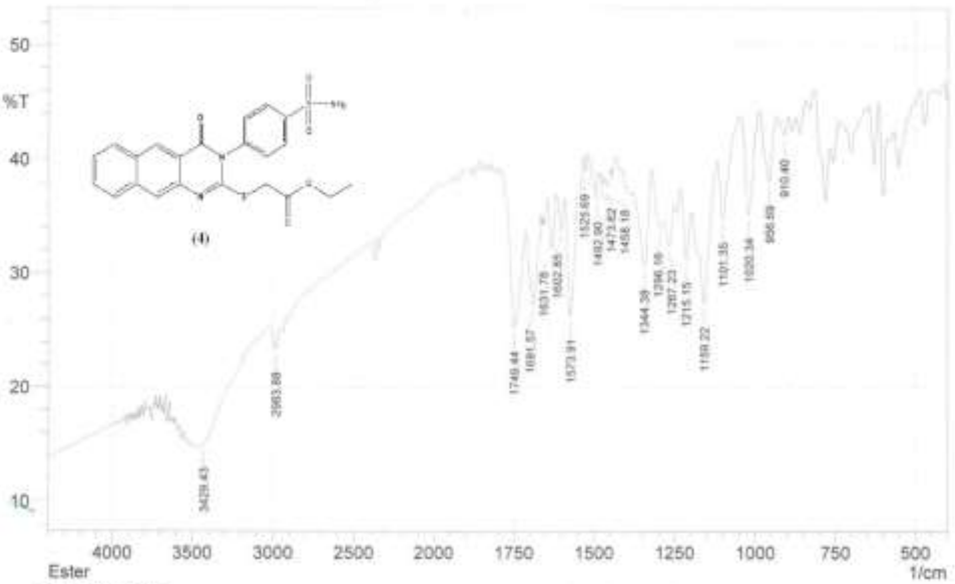

Date: 4/13/2016
Time: 2:25:53 PM

No. of Scans: 20
Resolution: 4

Peak	Intensity	Corr. Intensity	Base (H)	Base (L)	Area	Corr. Area
910.4	42.084	1.71	933.55	896.9	13.454	0.315
956.69	38.036	5.957	987.55	933.55	20.947	1.703
1020.34	35.165	8.842	1047.35	987.55	23.8	2.497
1101.35	34.77	5.971	1116.78	1047.35	27.995	1.686
1159.22	27.38	8.725	1176.58	1116.78	28.929	2.09
1215.15	31.31	5.094	1232.51	1197.79	16.305	1.063
1267.23	32.375	3.063	1284.59	1251.8	15.406	0.611
1296.16	33.882	2.09	1323.17	1284.59	17.273	0.529
1344.38	30.648	7.749	1379.1	1323.17	26.016	2.603
1458.18	37.657	0.85	1463.97	1448.54	6.466	0.092
1473.62	37.802	0.814	1479.4	1469.76	4.03	0.047
1492.9	36.589	2.522	1512.19	1485.19	11.345	0.394
1525.69	39.025	1.402	1533.41	1517.98	6.165	0.096
1573.91	26.344	10.396	1593.2	1548.84	22.26	2.985
1602.85	33.78	2.91	1616.35	1593.2	10.467	0.398
1631.78	32.215	4.496	1643.35	1616.35	12.529	0.785
1691.57	27.23	7.111	1712.79	1664.57	24.782	2.448
1749.44	25.274	1.69	1791.87	1745.58	22.428	-0.594
2983.88	23.369	1.942	3024.38	2951.09	44.837	1.113
3429.43	15.008	0.125	3433.29	3113.11	226.372	0.115

C:\Bruker\TOPSPI

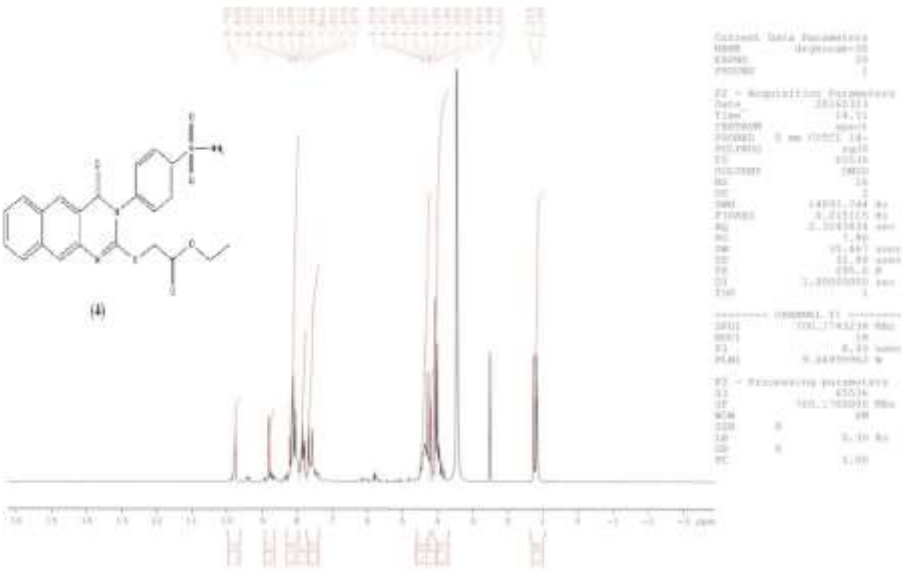

C13CPD DMSO (C:\Bruker\TOPSPIN) [illegible] 35

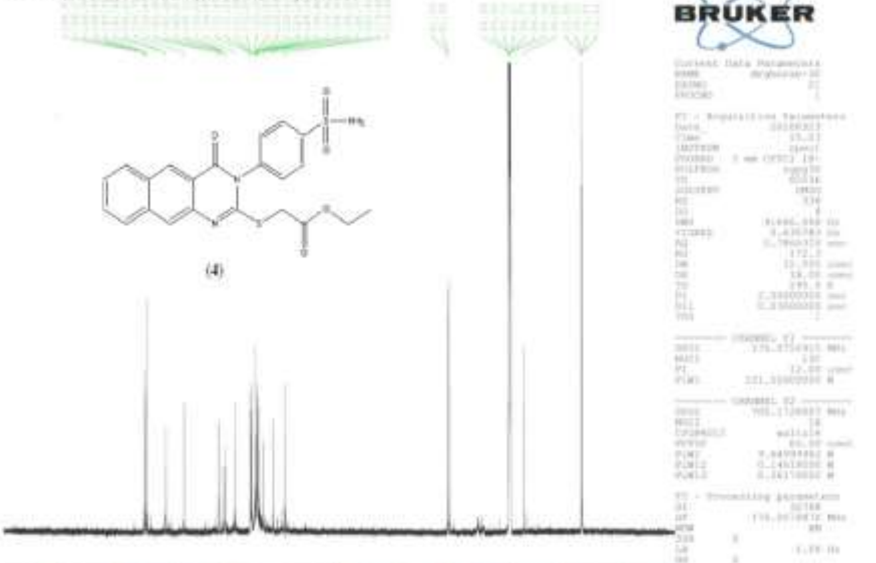

SHIMADZU

KSU - College of Pharmacy
Medicinal, Aromatic & Poisnous Plants Research Centre
FT- IR Report

Sample Name: Hydrazide stiring

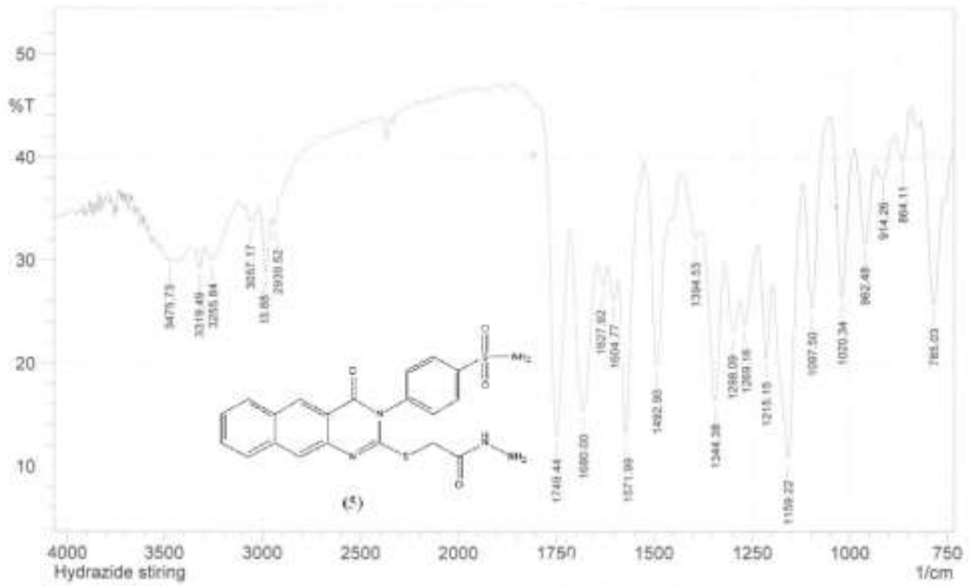

Date: 4/13/2016
Time: 2:46:06 PM

No. of Scans: 20
Resolution: 4

Peak	Intensity	Corr. Intensity	Base (H)	Base (L)	Area	Corr. Area
785.03	25.765	13.446	817.82	759.95	28.139	4.818
864.11	39.586	4.066	885.33	844.82	15.459	0.843
914.26	38.005	2.575	935.48	885.33	20.232	0.695
962.48	31.647	8.461	989.48	935.48	24.029	2.596
1020.34	26.432	15.964	1051.2	989.48	28.166	5.146
1097.5	25.4	14.277	1120.64	1051.2	31.846	4.674
1159.22	10.93	21.736	1197.79	1122.57	52.07	15.151
1215.15	20.506	8.989	1234.44	1197.79	22.028	2.616
1269.16	23.724	3.855	1282.66	1234.44	27.378	1.2
1298.09	23.115	4.534	1319.31	1282.66	21.826	1.5
1344.38	16.393	14.479	1382.96	1321.24	37.947	6.702
1394.53	32.091	2.038	1431.18	1382.96	22.053	0.376
1492.9	19.758	17.204	1523.76	1458.18	35.489	6.888
1571.99	13.095	18.195	1591.27	1525.69	38.748	7.494
1604.77	26.139	2.579	1618.28	1593.2	14.105	0.535
1627.92	27.508	2.015	1645.28	1618.28	14.674	0.377
1680	15.38	15.883	1712.79	1647.21	43.439	10.236
1749.44	13.023	25.053	1797.66	1714.72	47.211	12.935
2939.52	33.327	1.514	2954.95	2912.51	19.65	0.367
2983.88	28.873	5.705	3020.53	2954.95	32.386	2.194
3057.17	33.791	1.451	3086.11	3020.53	30.36	0.657
3255.84	30.042	2.22	3294.42	3120.82	85.079	2.423
3319.49	29.176	2.094	3361.93	3294.42	34.646	0.757
3475.73	29.91	0.07	3479.58	3464.15	8.082	0.01

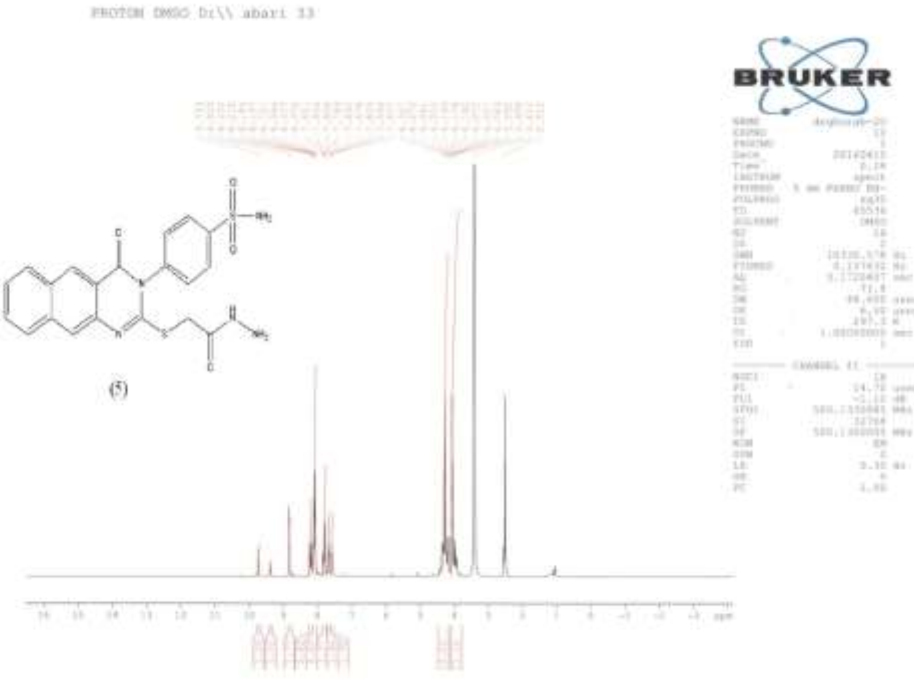
PROTON DMSO D:\\ abari 33
BRUKER
(5)

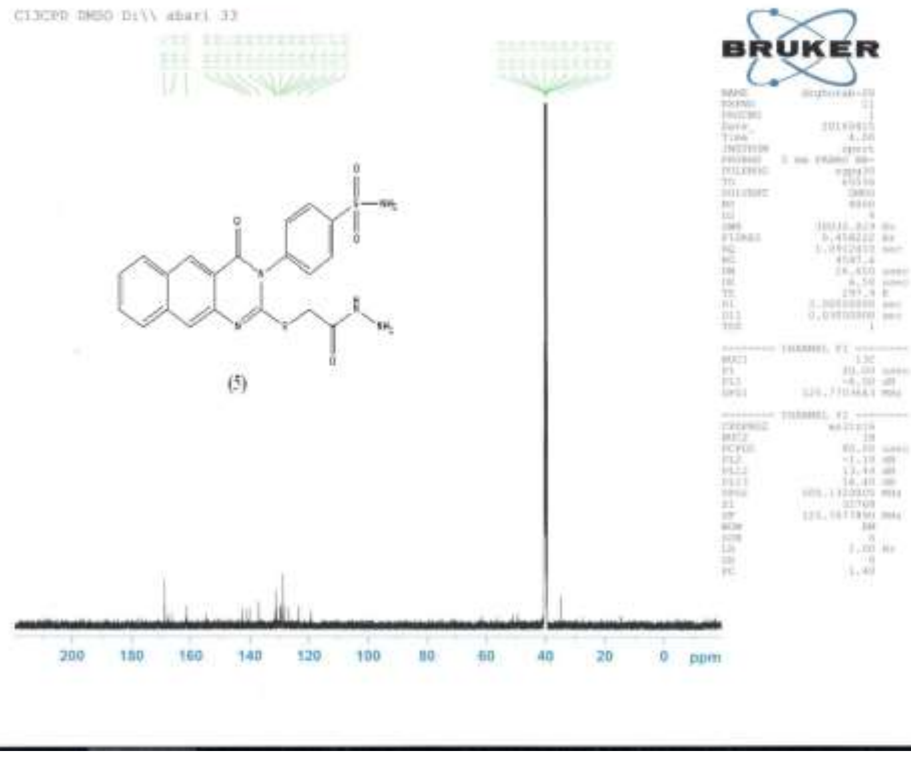
C13CPD DMSO D:\\ abari 33
BRUKER
(5)
200 180 160 140 120 100 80 60 40 20 0 ppm
